# Topical Application of Galgeunhwanggeumhwangryeon-Tang Recovers Skin-Lipid Barrier and Ameliorates Inflammation via Filaggrin-Thymic Stromal Lymphopoietin-Interleukin 4 Pathway

**DOI:** 10.3390/medicina57121387

**Published:** 2021-12-20

**Authors:** Sang-Hyun Ahn, Su Shin, Yoonju Do, Yunju Jo, Dongryeol Ryu, Ki-Tae Ha, Kibong Kim

**Affiliations:** 1 Department of Anatomy, College of Korean Medicine, Semyung University, Jecheon 27136, Korea; smkmana@semyung.ac.kr; 2 Department of Korean Medical Science, School of Korean Medicine, Pusan National University, Yangsan 50612, Korea; indiesonne@pusan.ac.kr (S.S.); uk0243@pusan.ac.kr (Y.D.); 3 Department of Molecular Cell Biology, Sungkyunkwan University School of Medicine, Suwon 16419, Korea; yj.eunice.jo@gmail.com (Y.J.); freefall@skku.edu (D.R.); 4 Department of Korean Pediatrics, School of Korean Medicine, Pusan National University, Yangsan 50612, Korea; 5 Department of Korean Pediatrics, Pusan National University Korean Medicine Hospital, Yangsan 50612, Korea

**Keywords:** Galgeunhwanggeumhwangryeon-tang, atopic dermatitis, ceramide, filaggrin, IL-4

## Abstract

*Background and objectives:* The purpose of this study was to confirm the effect of Galgeunhwanggeumhwangryeon-tang (GGRT) on the skin barrier integrity and inflammation in an atopic dermatitis-like animal model. *Materials and Methods:* The model was established using lipid barrier elimination (LBE) in BALB/c mice. Ceramide 3B, a control drug, and GGRT were applied to the skin of LBE mice. Gross observation and histological examination were combined with measurement of skin score, trans-epidermal water loss, and pH. The expression of filaggrin, kallikrein-related peptidase 7 (KLK7), protease-activated receptor-2 (PAR-2), thymic stromal lymphopoietin (TSLP), and interleukin 4 (IL-4) was examined. *Results:* The effect of GGRT on atopic dermatitis was estimated in silico using two individual gene sets of human atopic dermatitis. In animal experiments, GGRT treatment reduced atopic dermatitis-like symptoms, as confirmed via gross and histological observations, skin score, pH change, and trans-epidermal water loss. The expression level of filaggrin increased in the skin of GGRT-treated mice compared to that in the LBE group. The expression levels of KLK7, PAR2, TSLP, and IL-4 were decreased in GGRT-treated mice skin compared to those in LBE mice. *Conclusions:* We demonstrated that GGRT restored the skin barrier and reduced inflammatory reactions in a murine model of atopic dermatitis.

## 1. Introduction

Atopic dermatitis is a chronic inflammatory skin disease that originates from a complex interaction among various factors [1]. Atopic dermatitis is highly prevalent, affecting up to 20% of children and 1–3% of adults worldwide [2]. The disease generally classified to three types, i.e., persistent, relapsing, and adult-onset form. Despite many common features in these forms, there are significant difference between childhood-onset types and adult-onset type [3]. Patients with atopic dermatitis are at high risk of developing allergic rhinitis and asthma [1,2]. Several conventional drugs including steroid, calcineurin inhibitors, and moisturizing creams, are widely, used for treating atopic dermatitis, but often they are not effective in some patients [1,4]. Thus, novel drugs targeting specific molecules related to T cell regulation, inflammation, and barrier function are now under struggling [2,5].

The epidermis plays a critical role in preventing entry of allergens and microorganisms by providing a physical and functional barrier. Dysfunction of the skin barrier is the first step in the pathogenesis of atopic dermatitis [6]. Additionally, helper T 2 (Th2)-driven inflammation is a key factor in the development of the disease [7,8]. The strongest genetic predisposing factor to atopic dermatitis is loss-of-function mutations in the filaggrin gene, which is essential for skin barrier function [9,10]. Thus, filaggrin (a key skin barrier-related protein), as well as Th2-related factors, thymic stromal lymphopoietin (TSLP) and interleukin (IL)-4, are considered as potential therapeutic targets [7,11].

Galgeunhwanggeumhwangryeon-tang (GGRT), or gegen qinlian decoction in Chinese, is a traditional herbal formula according to Shanghan-lun and has been used to treat diarrhea accompanied to upper respiratory infection, which is common in children under 5 years [12]. The formula is composed of the roots of four medicinal plants, *Pueraria lobata* (Willd) Ohwi, *Scutellaria baicalensis* George, *Coptis japonica* Makino, and *Glycyrrhiza uralensis* Fischer [13]. Previous pharmacological studies have demonstrated that GGRT affects several diseases, such as ulcerative colitis, type 2 diabetes mellitus, and acute lung injury [14,15,16]. The modulation of the gut microbiota is estimated to be a key mechanism underlying the effects of GGRT on type 2 diabetes mellitus [17]. The herbal formula is one of the top 20 Shanghan formulae commonly used in traditional Chinese medicine office visits between 1999 and 2002, according to the National Health Insurance program of Taiwan [12]. Despite its frequent use and its effect on inflammatory diseases [12,14,15,16,17], the formula has not been used to treat skin disorders, including atopic dermatitis.

In this study, we aimed to investigate whether GGRT affects atopic dermatitis-like skin inflammation in BALB/c mice with compromised skin barrier. The mechanism underlying the anti-dermatitis effect of GGRT (skin barrier reconstruction and Th2-mediated immune response) was also elucidated. These results may open a new therapeutic avenue for the treatment of atopic dermatitis.

## 2. Materials and Methods

### 2.1. Materials

Primary antibodies, including mouse anti-filaggrin (1:100), mouse anti-kallikrein 7 (KLK7; 1:100), mouse anti-protease-activated receptor 2 (PAR-2; 1:100), and mouse anti-interleukin-4 (IL-4; 1:50), were purchased from Santa Cruz Biotechnology (Santa Cruz, CA, USA). Mouse anti-thymic stromal lymphopoietin (TSLP; 1:100) antibody was supplied by Abcam (Cambridge, UK). The standard compounds for identifying the herbal materials puerarin, daidzin, baicalin, wogonin, berberine, and palmatine, were supplied by the Ministry of Food and Drug Safety, Korean Government (MFDS; Cheongju, Korea). Glycyrrhizin was purchased from the Natural Product Bank of the National Development Institute of Korean Medicine (Gyeongsan, Korea). Other chemicals and reagents were purchased from Sigma-Aldrich (St. Louis, MO, USA), unless otherwise indicated.

### 2.2. Preparation of GGRT Extract

The composition of GGRT is shown in Table 1. The dried materials of the four medicinal herbs were purchased from Omniherb Co. (Daegu, Korea). The herbal materials were authenticated by the botanical expert of Omniherb Co. and were manufactured according to the herbal good manufacturing practice (hGMP) regulation controlled by MFDS. The specimens were kept at the School of Korean Medicine, Pusan National University (Yangsan, Korea). The dried GGRT material (150 g) was added to 2000 mL of distilled water, boiled for 3 h, and filtered. The filtrate was concentrated to 50 mL using a rotary evaporator (Eyela, Tokyo, Japan) and powdered using a freeze drier (Labconco, Kansas, MO, USA) to obtain 16.4 g of extract (yield: 10.9%). The GGRT extract was diluted in normal saline to prepare a 5% GGRT extract coating solution.

### 2.3. HPLC Analysis

The phytochemical properties of water extracted GGRT were identified by HPLC analysis. HPLC analysis was performed using an Agilent HPLC 1200 series system (Agilent Technologies, Santa Clara, CA, USA), and LC solution software was used to analyze the data. The ODS group C18 UG120 column (4.6 mm × 250 mm, 5 μm; Osaka Soda Co., Ltd., Osaka, Japan) was used as an analytical column. The mobile solvents were acetonitrile containing 0.1% formic acid (solvent A), and pure water containing 0.1% formic acid (solvent B); the gradient elution flow was A:B = 15:85 (0–15 min) → A:B = 22:78 (15–25 min) → A:B = 28:72 (25–40 min) → A:B = 45: 55 (40–41 min) → A:B = 15:85 (41–50 min) at a flow rate of 1 mL/min. The oven temperature was 40 °C, the ultraviolet detector wavelength was 265 nm, and the injection volume was 10 μL. GGRT extract (120.6 mg) was dissolved in 30 mL of methanol, sonicated, and filtered through a 0.45 μm membrane filter. Puerarin, daidzin, and berberine (0.2 mg/mL), 0.13 mg/mL of palmatine, 0.44 mg/mL of baicalin, 0.26 mg/mL of wogonin, and 0.4 mg/mL of glycyrrhizin were prepared as standard compounds, according to previous studies [18,19,20,21].

### 2.4. Bioinformatic Analysis

The Kyoto Encyclopedia of Genes and Genomes (KEGG) pathway analysis was performed using publicly available transcriptomes of human atopic dermatitis tissues as described previously [22]. Two independent transcriptomes, GSE157194 [23] and GSE140227, are available at the Gene Expression Omnibus (GEO) of the National Center for Biotechnology Information (NCBI). Gene set enrichment analysis (GSEA; version 4.1.0) and Cytoscape (version 3.8.2) were used to conduct network analysis of the KEGG pathways. Gene network analysis presenting the pattern of co-expression in each group was generated based on Spearman’s correlation. The visualization of Gene Network was conducted using RStudio (RStudio Desktop v1.4.1717 with R v4.1.1), as previously described [24].

### 2.5. Animals

Four-week-old male BALB/c mice (OrientBio, Seongnam, Korea) were acclimatized for 2 weeks in an aseptic breeding apparatus, and then mice weighing 20 ± 1.0 g were selected and used. Mice were maintained in the animal room at 23–25 °C, 55 ± 10% relative humidity, and a 12 h light/dark cycle. A standard pellet diet and filtered tap water were provided ad libitum. Animal experiments were conducted after the approval of the Animal Experimental Ethics Committee of Pusan National University (IACUC No. PNU-2015-0924). The care and use of animals were conducted according to the NIH guidelines.

### 2.6. Induction of Model and Drug Treatment

Experimental animals were divided into four groups, including the control group, lipid barrier elimination group (LBE), ceramide 3B applied group after lipid barrier elimination (C3A), and GGRT extract-treated group after lipid barrier removal (GGRT). Ten animals were assigned to each group. After shaving the dorsal skin of the mice using a depilatory cream (Body Natur, Nueil-les-Aubiers, France), the stratum corneum (desquamation) was removed using tape (3M, St. Paul, MN, USA). After applying 500 μL of 10% sodium dodecyl sulfate (Sigma-Aldrich), the lipid lamella of the stratum corneum was removed by rubbing it 20 times using a cotton swab. Then, 100 μL of 5% GGRT extract dissolved in isotonic sodium chloride solution was applied to the lipid lamella-removed skin of mice in the GGRT group for 3 days. Isotonic sodium chloride solution (100 μL) and 5% ceramide 3B (Ecofactory, Incheon, Korea) in isotonic sodium chloride solution were used as negative and positive control drugs, respectively, and were applied to C3A group mice for 3 days after removing the lipid lamella.

### 2.7. Evaluation of Skin Dermatitis Severity

The severity of morphology in the dorsal skin was evaluated after 3 weeks and compared with the baseline. The skin score items were (1) erythema/hemorrhage, (2) scarring/dryness, (3) edema, and (4) excoriation/erosion was scored as 0 (none), 1 (mild), 2 (moderate), or 3 (severe). The sum of the individual scores was defined as the atopic skin score [25].

### 2.8. TEWL and pH Measurement

Three days after the removal of the fat barrier, trans-epidermal water loss (TEWL), and changes in skin pH were measured. TEWL was measured with a vapometer (Delfin Technologies, Kuopio, Finland), and skin pH changes were measured using Skin-O-Mat (SM815, CK Electronics, Cologne, Germany).

### 2.9. Tissue Chemistry

Cardiac perfusion fixation was performed on the skin with a vascular rinse and 10% neutral buffered formalin (NBF). After the obtained dorsal skin was fixed in 10% NBF for 24 h, it was embedded in paraffin using a conventional method, and serial sections were made with a thickness of 5 μm. Serial sections were observed after staining with hematoxylin and eosin.

### 2.10. Immunohistochemistry

For immunohistochemical staining, antibodies against filaggrin, KLK7, PAR-2, TSLP, and IL-4 were used. First, the skin sections were subjected to proteolysis in proteinase K (20 μg/μL; Agilent Dako, Santa Clara, CA, USA) for 5 min and then treated with 10% normal goat serum (Vector Lab, Burlingame, CA, USA) containing 1% fetal bovine serum (Sigma-Aldrich) for 1 h. Then, the appropriate primary antibody was reacted in a humidified chamber at 4 °C for 72 h. The secondary antibody, biotinylated goat anti-mouse IgG (1:100, Abcam), was linked for 24 h at room temperature and reacted with an avidin-biotin complex kit (Vector Lab) for 1 h at room temperature. After color development in 0.05 M Tris-HCl buffer (pH 7.4) containing 0.05% 3,3′-diaminobenzidine and 0.01% HCl, counterstaining was performed with hematoxylin.

### 2.11. Image Analysis

The results of immunohistochemistry were quantified by image analysis using Image Pro 10 (Media Cybernetics, Rockville, MD, USA). After randomly selecting 10 skin samples from each group, they were photographed at ×400 magnification, and then images were analyzed with positive pixels (intensity 80–100)/2 × 10^7^ pixels.

### 2.12. Statistical Analysis

For statistical analysis, the experimental data were analyzed using SPSS software (SPSS 25, SPSS Inc., Chicago, IL, USA). All results are expressed as mean ± standard deviation (SD). The statistically significant differences were verified using one-way analysis of variance (ANOVA) and Tukey’s post-hoc test. Statistical significance was set at *p* < 0.05.

## 3. Results

### 3.1. Identification of GGRT Extract by HPLC Analysis

**Figure 1:**
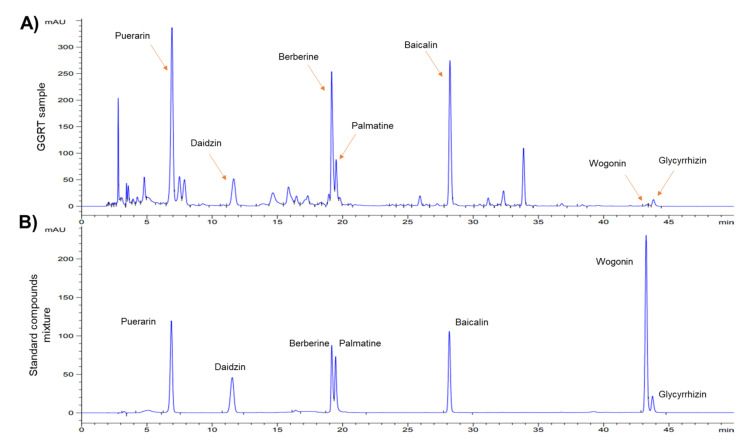
High-performance liquid chromatography (HPLC) fingerprinting analysis of GGRT extract. (**A**) HPLC chromatogram of GGRT was monitored with an ultraviolet detector of 265 nm wavelength. (**B**) The mixture of standard compounds was also analyzed under the same conditions as GGRT.

To evaluate the phytochemical properties of the GGRT extract, high-performance liquid chromatography (HPLC) analysis was conducted using water extracted GGRT and a mixture of standard compounds. Seven previously known compounds, such as puerarin, daidzin, berberine, palmatine, baicalin, wogonin, and glycyrrhizin [18,19,20,21], which were found in the four herbal medicines in GGRT, were confirmed (Figure 1).

### 3.2. Human Transcriptomic Analysis Indicates GGRT as a Therapeutic for Atopic Dermatitis

**Figure 2:**
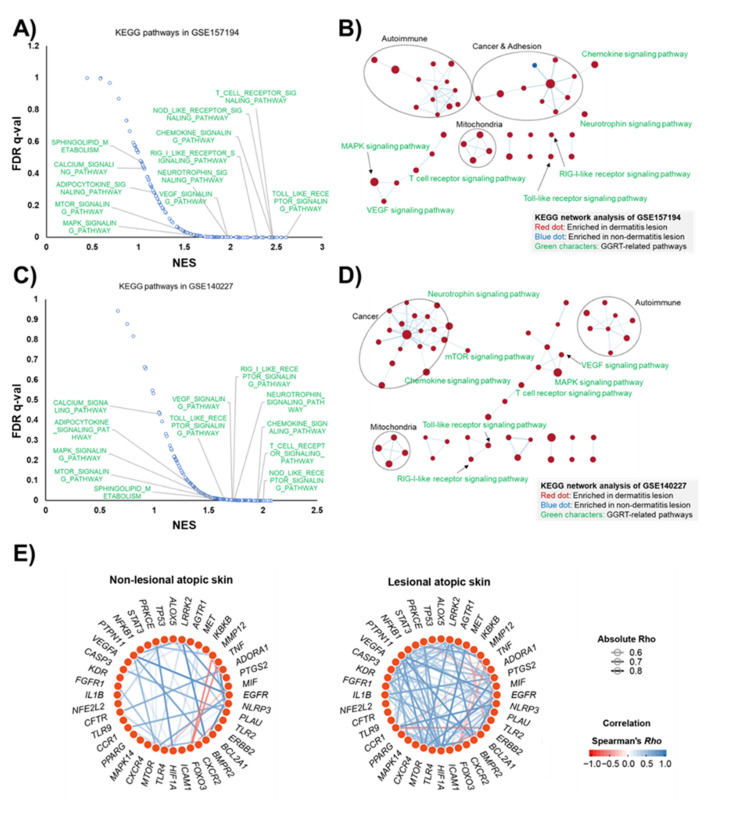
In silico prediction for GGRT treatment of atopic dermatitis. (**A**,**C**) The enhanced pathways of GSEA KEGG enrichment analysis for potential targets of GGRT in two individual gene sets (GSE157194 and GSE140227). (**B**,**D**) The potential targets of GGRT were overlapped on the KEGG network analysis of two individual gene sets (GSE157194 and GSE140227). Red dot represent enriched pathways in atopic dermatitis lesions and the blue dots represent enriched pathways in non-disease lesions. Green characters mean the pathways are related to GGRT. (**E**) Gene networks in non-lesional and lesional skins from atopic dermatitis patients were generated based on correlations (Spearman’s Rho > |0.6| and *p* < 0.05) among genes, known to mediate the mechanism of action of GGRT. Each edge represents Spearman’s Rho of two connected genes. The red or blue gradient color of each edge indicates a positive or negative correlation, respectively.

According to a recent systems pharmacological study that revealed the mechanism of action (MOA) of GGRT using a multi-omics approach, including transcriptomic KEGG enrichment and metabolomic analyses [16], the targets corresponding to each component of GGRT were related to inflammatory signaling pathways, such as NF-κB, TNF-α, HIF-1, Toll-like receptor, NOD-like receptor, adipocytokine, and chemokines (summarized in Supplementary Figure S1). We conducted KEGG enrichment analysis using two different transcriptomes from 57 or 6 human atopic dermatitis patients (GSE157194 and GSE140227, available at the NCBI GEO). The results showed that 12 signaling pathways, Toll-like receptor, T cell receptor, nucleotide-binding oligomerization domain (NOD)-like receptor, retinoic acid-inducible gene I (RIG-I)-like receptor, chemokine, neurotrophin, vascular endothelial cell growth factor (VEGF), sphingolipids, calcium, adipocytokine, mammalian target of rapamycin (mTOR), and mitogen-activated protein kinase (MAPK), among 22 of the predicted GGRT-related pathways were commonly upregulated in the atopic dermatitis lesion (Figure 2A,C). Detailed information of the KEGG enrichment analysis is shown in Supplementary Tables S1 and S2. KEGG network analysis revealed that the two independent gene sets showed very similar network patterns, and seven signaling pathways among GGRT-related KEGG pathways (chemokine, neurotrophin, RIG-I-like receptor, Toll-like receptor, T cell receptor, MAPK, and VEGF), were commonly upregulated in the two independent human atopic dermal transcriptomes (Figure 2B,D). In addition to GGRT-related pathways, signaling pathways associated with autoimmunity, mitochondria, and cancer were also upregulated in both KEGG network analyses. Furthermore, gene network analysis based on Spearman’s correlation revealed that genes encoding the previously proposed factors [16] mediating the MOA of GGRT were tightly correlated with each other and generated a co-expressing gene network in the lesions of atopic dermatitis (Figure 2E). Interestingly, the number of correlated genes, which were visualized with edges, was increased in skin lesions of atopic dermatitis patients compared to non-lesional sites. These results of human atopic dermal transcriptomic analysis led us to hypothesize that GGRT is a possible therapeutic candidate for atopic dermatitis.

### 3.3. GGRT Restores the Skin Lipid Barrier

**Figure 3:**
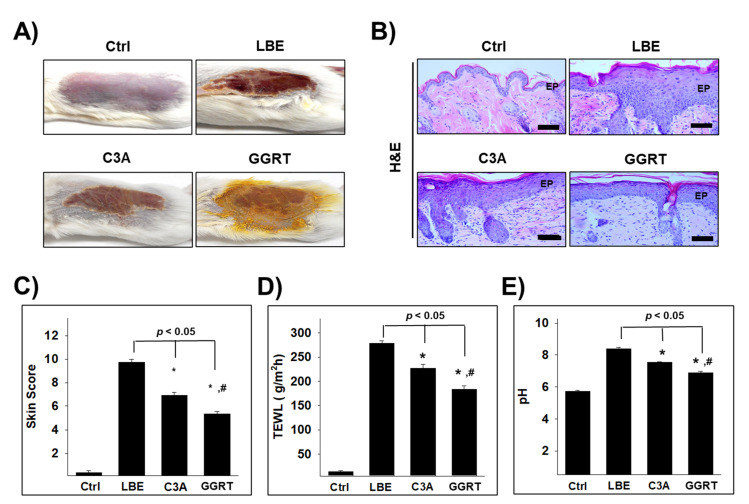
The effects of GGRT on lipid barrier elimination-induced atopic dermatitis lesions. The atopic dermatitis was induced by the elimination of the lipid barrier by SDS application. Isotonic sodium chloride solution, 5% ceramide, and 5% GGRT were administrated to lipid-eliminated mice skins for 3 days. (**A**) Photographs of skins were taken for macroscopic observation. (**B**) The fixed skins underwent H&E staining for histopathological examination. Bar size, 50 μm. (**C**–**E**) Skin score, TEWL, and pH were examined. *, *p* < 0.05 compared with LBE; #, *p* < 0.05 compared with C3A. Abbreviations: Ctrl, healthy control group; LBE, normal saline-treated group after lipid barrier elimination; C3A, 5% ceramide 3B treated group after lipid barrier elimination; GGRT, 5% GGRT extract treated group after lipid barrier elimination; TEWL, trans-epidermal water loss; H&E, hematoxylin and eosin; EP, epithelium.

To validate this hypothesis, we evaluated the effects of GGRT in rodent models of dermatitis. First, a histological analysis was performed. After the fat barrier was removed, macroscopic observation of the skin in the lipid barrier elimination (LBE) group demonstrated severe dermatitis signs, including scarring at the edge boundary, and erythema, hemorrhage, and erosion in the center of the excoriated skin. In ceramide 3B (C3A) and GGRT-treated mice, the dermatitis symptoms and size of the lesion were reduced compared to those in the LBE group (Figure 3A). Histochemical findings showed structural changes, such as epithelial cell hyperplasia, expansion of the intercellular space of the spinous layer, increased lymphocyte infiltration, and collapse of the basal layer, in LBE mice. These changes were reduced in the skin of the C3A and GGRT groups. The structural damage was more markedly recovered in the GGRT group than in the C3A group (Figure 3B).

The skin score was elevated in the LBE group (9.7 ± 0.27) compared to that in the control group (0.4 ± 0.15), whereas it was significantly reduced in the C3A group (6.9 ± 0.25) and the GGRT group (5.4 ± 0.2) (Figure 3C). Trans-epidermal water loss (TEWL) increased approximately 19-fold in the LBE group (278 ± 6 g/m^2^h) than in the control group (14 ± 1.4 g/m^2^h). In the C3A (227 ± 7 g/m^2^h) and GGRT (183 ± 8 g/m^2^h) groups, the TEWL was reduced 15-fold and 12-fold from the LBE group, respectively (Figure 3D). The skin pH also increased in the LBE (8.38 ± 0.09) group from that in the control group (5.72 ± 0.05). The elevated pH in the LBE group was markedly reduced in the C3A (7.54 ± 0.04) and GGRT (6.88 ± 0.08) groups (Figure 3E).

### 3.4. GGRT Recovers the Skin Barrier-Related Proteins

**Figure 4:**
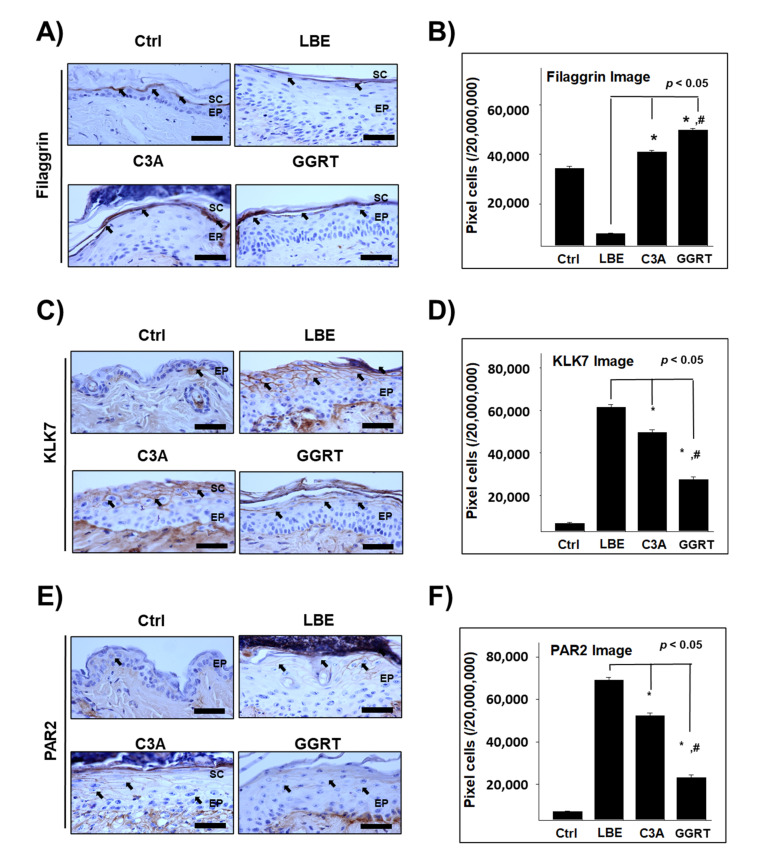
The effect of GGRT on the expression of skin barrier-related proteins. Atopic dermatitis was induced by elimination of the lipid barrier via SDS application. Isotonic sodium chloride solution, 5% ceramide, and 5% GGRT were administrated to lipid-eliminated mice skins for 3 days. (**A**,**C**,**E**) Filaggrin, KLK7, and PAR-2 (arrows indicate light brown particle) were visualized by immunohistochemistry using corresponding antibodies. Bar size, 50 μm. (**B**,**D**,**E**) The densitometric data is shown as positively stained cells per 2 × 10^7^ pixels of images for each protein. *, *p* < 0.05 compared with LBE; #, *p* < 0.05 compared with C3A. Abbreviations: Ctrl, healthy control group; LBE, normal saline-treated group after lipid barrier elimination; C3A, 5% ceramide 3B treated group after lipid barrier elimination; GGRT, 5% GGRT extract treated group after lipid barrier elimination; SC, stratum corneum; EP, epithelium.

The expression level of filaggrin, a key regulator of the skin barrier in atopic dermatitis [26], in the LBE group (7962 ± 328/2 × 10^7^ pixels) was significantly lower than that in the control group (34,276 ± 818/2 × 10^7^ pixels). Conversely, filaggrin expression levels in the C3A (41,039 ± 622/2 × 10^7^ pixels) and GGRT (49,842 ± 557/2 × 10^7^ pixels) groups were significantly higher than that in the LBE group (Figure 4A,B). The expression level of kallikrein-related peptidase 7 (KLK7), an atopic dermatitis-associated serine protease [27], was elevated approximately 8-fold in the LBE group (61,577 ± 1080/2 × 10^7^ pixels) compared to that of the control group (6771 ± 399/2 × 10^7^ pixels). In C3A (49,673 ± 1133/2 × 10^7^ pixels) and GGRT (27,420 ± 1183/2 × 10^7^ pixels) groups, the KLK7 expression level was significantly reduced from that in the LBE group (Figure 4C,D). The expression level of protease-activated receptor-2 (PAR-2), a key receptor regulating inflammation and ichthyosis in barrier-damaged skin [28,29], was higher in the LBE group (69,165 ± 1235/2 × 10^7^ pixels) by approximately 8.7-fold compared to that in the control group (7084 ± 359/2 × 10^7^ pixels). Compared to that in the LBE group, the PAP-2 expression levels in C3A (52,380 ± 1189/2 × 10^7^ pixels) and GGRT (23,217 ± 1245/2 × 10^7^ pixels) groups were 76% and 34% lower, respectively (Figure 4E,F). From these results, GGRT was more effective at recovering the expression of skin barrier-related proteins compared to the control drug, C3A.

### 3.5. GGRT Reduces the Th2-Related Inflammatory Factors

**Figure 5:**
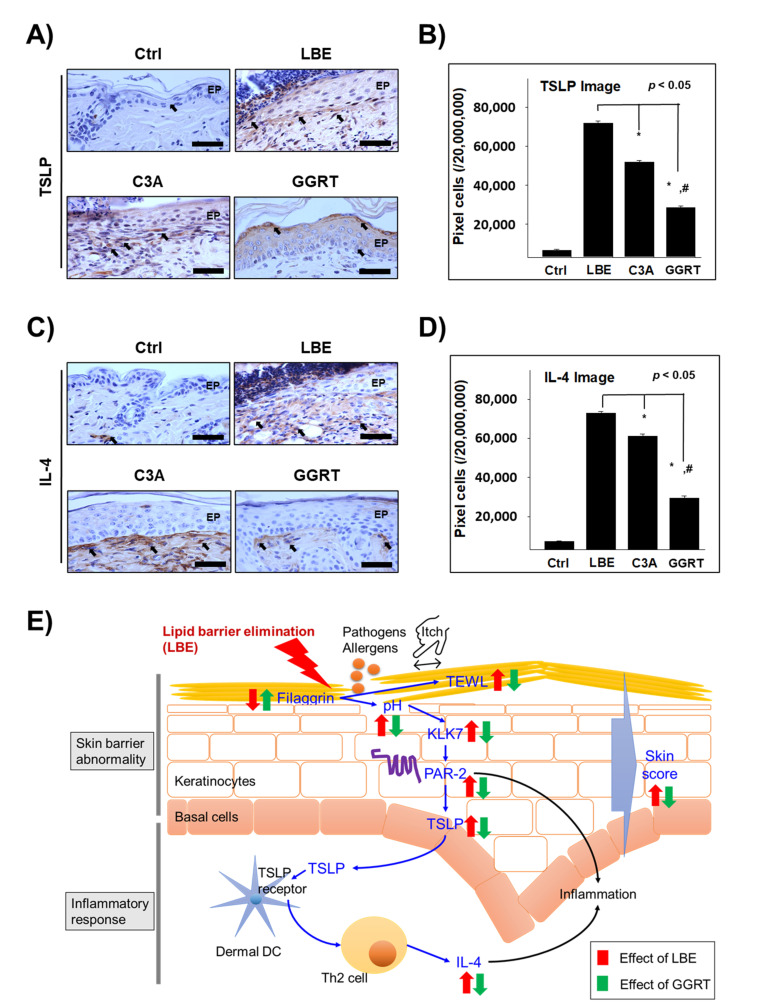
The effect of GGRT on the regulation of Th2 differentiation-related proteins. Atopic dermatitis was induced by the elimination of the lipid barrier via SDS application. Isotonic sodium chloride solution, 5% ceramide, and 5% GGRT were administered to lipid-eliminated mice skins for 3 days. (**A**,**C**) TSLP and IL-4 (arrows indicate light brown particle) were visualized by immunohistochemistry using corresponding antibody. Bar size, 50 μm. (**B**,**D**) The densitometric data is shown as positively stained cells per 2 × 10^7^ pixels of images for each protein. *, *p* < 0.05 compared with LBE; #, *p* < 0.05 compared with C3A. (**E**) Schematic representation of inhibition of GGRT on the LBE-induced atopic-like dermatitis. Abbreviations: Ctrl, healthy control group; LBE, normal saline-treated group after lipid barrier elimination; C3A, 5% ceramide 3B treated group after lipid barrier elimination; GGRT, 5% GGRT extract treated group after lipid barrier elimination; EP, epithelium.

The expression level of TSLP, a key factor for promoting Th2 response in atopic dermatitis [30], was elevated by approximately 9.8-fold in the skin of LBE group mice (71,974 ± 1066/2 × 10^7^ pixels) than in the skin of control group mice (6659 ± 343/2 × 10^7^ pixels). In the C3A (52,004 ± 872/2 × 10^7^ pixels) and GGRT (28,621 ± 793/2 × 10^7^ pixels) groups, TSLP expression level was significantly reduced to 72% and 40% compared to the LBE group, respectively (Figure 5A,B). The expression level of IL-4, a representative Th2-skewing cytokine [31], was also strongly elevated in the dermal papilla of LBE group mice (72,845 ± 954/2 × 10^7^ pixels) compared to that of the control group mice (7164 ± 371/2 × 10^7^ pixels), up to approximately 9-fold. IL-4 expression levels in the C3A (61,106 ± 1139/2 × 10^7^ pixels) and GGRT (29,596 ± 987/2 × 10^7^ pixels) groups were markedly decreased to 84% and 41% compared to those in the LBE group, respectively (Figure 5C,D). The results demonstrated that the effect of GGRT on the expression of Th2-related proteins was more prominent than that of the positive control, C3A.

## 4. Discussion

Symptoms of atopic dermatitis, such as itchy, dry skin, and eczema erythematosus, are caused by damaged skin barriers [6,11,32]. The epidermis functions a protective barrier by forming a stratum corneum structure composed of corneocytes, cornified envelopes, lamellar membrane lipids, intercorneocyte lipids, and corneodesmosomes [33]. The cornified protein envelope of keratinocytes, comprising proteins such as involucrin, loricrin, and trichohyalin, is crucial for maintaining the integrity of the physical barrier [34,35]. In the stratum corneum of patients with atopic dermatitis, levels of lipids (including ceramide), which function as a barrier, and retained water are diminished [36,37,38]. Therefore, minimizing and recovering damage to the skin barrier has been the focus of research to develop therapeutics for preventing or treating atopic dermatitis [6,11]. Thus, in this study, we focused on the effect of herbal formulas on the recovery of the skin barrier using a lipid barrier-damaged mouse model.

Filaggrin, a filament-associated protein, connects the outer keratinocytes and keratin, and firmly adheres and fixes keratin in the stratum corneum [39]. It also maintains the skin barrier by binding proteins such as involucrin, loricrin, and keratin [6,40]. Filaggrin loss-of-function mutations cause skin barrier damage and IgE sensitization in patients with atopic dermatitis [10,41,42]. Filaggrin is also decomposed by enzymes and thereby binds with water to act as a moisturizing factor [43]. It also plays other protective roles, such as pH control and UV filtration, in the stratum corneum [26,42]. In normal skin tissues, proteolytic enzymes involved in the exfoliation of keratinocytes are tightly regulated by the pH of the stratum corneum [3]. In atopic dermatitis patients, the pH of the atopic lesions is generally higher than that of healthy skin [44].

When the pH increases in the stratum corneum of patients with atopic dermatitis for various reasons, the activity of serine proteases, including kallikrein 5 protease, is markedly increased and destroys the skin barrier in the murine atopic dermatitis model [45]. The expression level of KLK7, a physiological activator of caspase 14, and the enzyme initiating the degradation of filaggrin, is mostly increased in human and murine atopic dermatitis tissues [27,46]. A series of proteolytic processes triggers the activation of PAR-2, a type of G protein-coupled receptor that senses cleaved amino-terminus small peptides, and consequently causes Th2 inflammation and skin pruritus [28,47]. The activation of PAR-2 stimulates the homing of Th2 cells by increasing the expression levels of Th2-related cytokines, such as TSLP and IL-4 [30,48]. Because PAR-2 inactivation directly reduces the expression level of TSLP, PAR-2 has been regarded as a therapeutic target for treating atopic dermatitis [49,50]. In addition, TSLP plays a critical role in the production of IL-4 from Th2 cells in atopic dermatitis through crosstalk between epithelial cells and dermal dendritic cells [30,51]. Thus, secretion of IL-4, a typical cytokine related to Th2 cells, induces excessive inflammation through several pathways, including secretion of IgE and activation of the Fc ε receptor [31,52,53].

In the present study, elimination of the lipid barrier increased skin pH, TEWL, and skin score. The expression level of filaggrin, a barrier-forming protein, was reduced by lipid barrier deprivation, whereas the expression levels of KLK7, PAR-2, TSLP, and IL-4 were significantly increased. Treatment with C3A and GGRT effectively reduced the indices of atopic dermatitis, such as pH, TEWL, and skin score. In addition, the expression level of filaggrin was reduced by C3A and GGRT treatment, and the expression levels of dermatitis-related proteins, including KLK7, PAR-2, TSLP, and IL-4, were markedly recovered. Furthermore, GGRT recovered the expression of skin barrier- and dermatitis-related proteins more effectively than the control drug, C3A. These results demonstrate that the GGRT extract reduced atopic dermatitis-like symptoms by recovering skin barrier-related proteins and suppressing Th2-related inflammation. However, in this study, we could not specify the precise mode of action (MoA) underlying the ameliorating effect of GGRT on barrier elimination-induced model of atopic dermatitis. As herbal formulas are composed of several different herbal medicines and thus have complex components, it is difficult to identify the direct molecular targets or precise pathways. To elucidate the MoA, further extensive studies examining the active compounds and their molecular targets are required.

In traditional medicine in Eastern Asia, atopic dermatitis is considered to originate from fetal heat, a group of diseases with heat manifestations occurring in the newborn due to the contraction of heat toxin in the fetal stage [54,55]. Thus, herbal medicines that produce a cooling effect are widely used clinically for the treatment of atopic dermatitis [56]. Although GGRT has not been clinically used to treat atopic dermatitis, herbal medicines, such as *P. lobata*, *S. baicalensis*, *C. japonica*, and *G. uralensis*, and their active compounds, including puerarin, licoricidin, and magnoflorine, were previously considered as candidates for treating atopic dermatitis [56,57,58,59,60,61,62]. In addition, previous experimental and clinical studies on GGRT revealed that the herbal formula has a pharmacological effect on diabetes mellitus, dyslipidemia, ulcerative colitis, and acute lung injury [14,15,16,17,63,64,65]. The mechanisms underlying the effect of GGRT on these diseases were estimated and evaluated by several in silico multi-omics and network pharmacological studies [14,15,16,17,64,66,67]. From these previous studies on the ingredients and in silico studies, we estimated that GGRT might have a potent effect on atopic dermatitis and evaluated its effectiveness using the lipid barrier elimination model in mice.

Furthermore, the ingredient compounds contained in GGRT are previously known as possible anti-atopic dermatitis. Puerarin reduced the atopic dermatitis-like skin lesion through suppressing inflammatory responses [59]. A metabolite of diadzin, 7,8,4′-Trihydroxyisoflavone also ameliorates the 2,4-dinitrochlorobenzene-induced atopic dermatitis-like symptoms and pro-inflammatory cytokines [59]. Wogonin also increased the antioxidant gene, heme oxygenase 1 and reduced mite antigen-induced thymus- and activation-regulated chemokine expression in human keratinocyte [68]. Berberine showed anti-atopic dermatitis effect through reducing cutaneous eukaryotic translation initiation factor 3-mucosa-associated lymphoid tissue lymphoma translocation protein 1 pathway [69]. The MoA of these natural products is not corresponding to protection of skin barrier. However, several studies showed that these compounds, including puerarin, berberine, and wogonin, regulated the mammalian target of rapamycin (mTOR) and signal transducer and activator of transcription 3 (STAT3) [70,71,72,73,74,75]. The mTOR and STAT3 signaling pathways have been known as crucial for maintaining homeostasis of skin barrier in pathophysiology of atopic dermatitis [76,77]. Thus, we postulate that these mechanisms might be involved in the anti-atopic dermatitis effect of GGRT or their ingredient. To assess the possibility, the further extensive studies are needed.

In this study, we first confirmed that GGRT influences skin barrier recovery and exerts anti-inflammatory effects on atopic dermatitis. The effect of GGRT on dermatitis was much greater than that of the control drug, ceramide, in terms of skin barrier reconstitution and inflammation relief. However, this study has several limitations, including safety concerns and application route. Although GGRT is generally administered orally in clinical settings, we applied the herbal formula topically. The formula has been orally administered up to 62 g for 8 weeks in several clinical studies [78]. Thus, we assume that the dose used in this study, 5% in normal saline, is too low to induce any safety issues, because the dermal absorption rate is much lower than that in the gastrointestinal tract [79]. In addition, since this study is limited to animal experiments, it only suggests the possibility of efficacy in humans. To confirm the clinical efficacy and safety, further studies and clinical trials are needed.

## 5. Conclusions

In this study, we evaluated the therapeutic efficacy of GGRT extract on an atopic dermatitis-like damaged skin model. GGRT recovered the damaged skin as confirmed by gross examination, histopathological observation, and measurement of TEWL and pH. Proteins related to skin barrier structure, such as filaggrin, KLK7, and PAR-2, were recovered in GGRT-treated mice. In addition, the LBE-stimulated expression levels of TSLP and IL-4, key regulators of Th2-related inflammation, were significantly decreased by GGRT treatment. From these results, we suggest that topical application of GGRT extract on skin with damaged lipid barrier might help recover the skin barrier integrity and relieve the inflammatory response.

## Figures and Tables

**Table 1:** Composition of galgeunhwanggeumhwangryeon-tang (GGRT).

Scientific Name	Botanical Name	Marker Compounds	Amount (g)
*Pueraria lobata* (Willd.) Ohwi	Puerariae Radix	Puerarin, Daidzin	80
*Scutellaria baicalensis* George	Scutellaria Radix	Baicalin, Wogonin	20
*Coptis japonica* Makino	Coptidis Rhizoma	Berberine, Palmatine	30
*Glycyrrhiza uralensis* Fischer	Glycyrrhiza Radix	Glycyrrhizin	20
Total			150

## Data Availability

The data will be made available upon reasonable request.

## Supplementary Materials

The following are available online at https://www.mdpi.com/article/10.3390/medicina57121387/s1. Figure S1: The KEGG signaling pathways predicted as potential GGRT targets. Table S1: KEGG enrichment analysis of GSE157194. Table S2: KEGG enrichment analysis of GSE140227.
