# Supplementary material for: Topical Application of Galgeunhwanggeumhwangryeon-Tang Recovers Skin-Lipid Barrier and Ameliorates Inflammation via Filaggrin-Thymic Stromal Lymphopoietin-Interleukin 4 Pathway
Source: Medicina (Kaunas). 2021 Dec 20;57(12):1387. doi: 10.3390/medicina57121387 (PMC8708970; doi:10.3390/medicina57121387)
Supplement: Supplementary file 1 [file medicina-57-01387-s001.zip › medicina-1488857-supplementary.pdf]

Supplementary materials

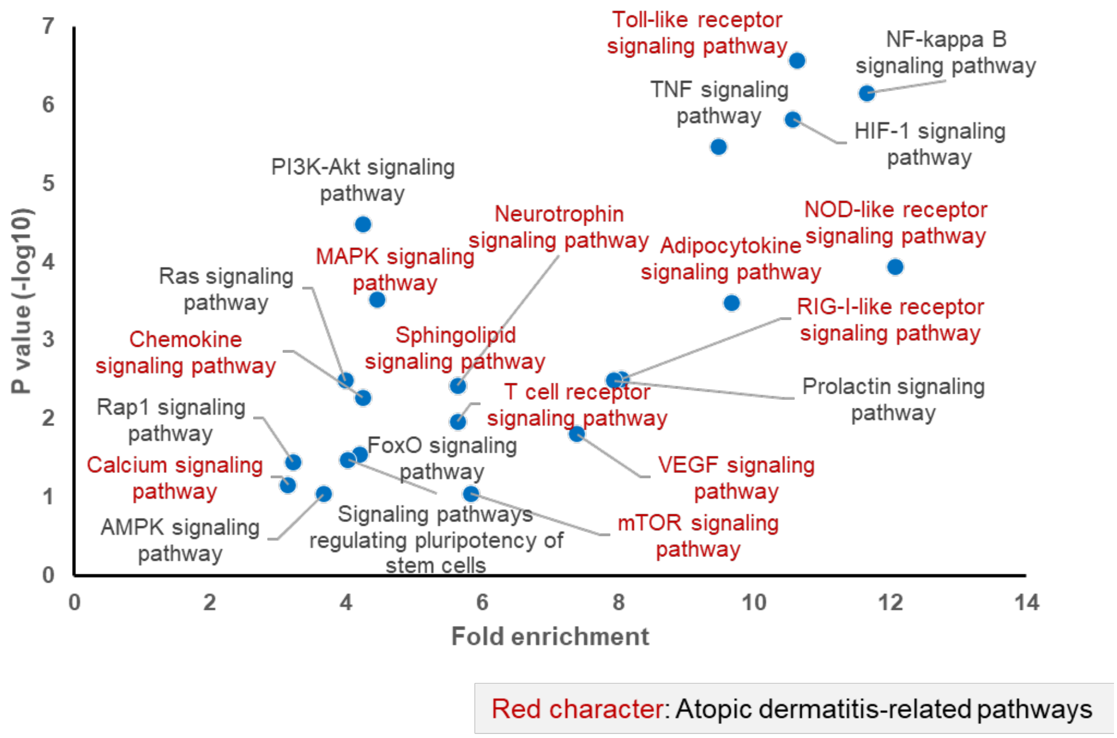

Supplementary Figure 1. The KEGG signaling pathways predicted as potential GGRT targets. The network pharmacologic analysis was shown by their fold enrichment and p values according to Ding *et al* [1]. Red characters mean the signaling pathways related to atopic dermatitis determined by GSEA KEGG analysis using two individual gene sets, GSE157194 and GSE140227.

1. Ding, Z.; Zhong, R.; Yang, Y.; Xia, T.; Wang, W.; Wang, Y.; Xing, N.; Luo, Y.; Li, S.; Shang, L., et al. Systems pharmacology reveals the mechanism of activity of Ge-Gen-Qin-Lian decoction against LPS-induced acute lung injury: A novel strategy for exploring active components and effective mechanism of TCM formulae. Pharmacol Res 2020, 156, 104759, doi:10.1016/j.phrs.2020.104759.
